# Methods for Examining Electrophysiological Coherence in Epileptic Networks

**DOI:** 10.3389/fneur.2013.00055

**Published:** 2013-05-15

**Authors:** Jasmine Song, Don M. Tucker, Tara Gilbert, Jidong Hou, Chelsea Mattson, Phan Luu, Mark D. Holmes

**Affiliations:** ^1^Electrical Geodesics, Inc.Eugene, OR, USA; ^2^Department of Psychology, University of OregonEugene, OR, USA; ^3^Department of Neurology, Regional Epilepsy Center, University of WashingtonSeattle, WA, USA

**Keywords:** epilepsy, spike, coherence, networks, dEEG, cortical surface Laplacian

## Abstract

Epilepsy may reflect a focal abnormality of cerebral tissue, but the generation of seizures typically involves propagation of abnormal activity through cerebral networks. We examined epileptiform discharges (spikes) with dense array electroencephalography (dEEG) in five patients to search for the possible engagement of pathological networks. Source analysis was conducted with individual electrical head models for each patient, including sensor position measurement for registration with MRI with geodesic photogrammetry; tissue segmentation and skull conductivity modeling with an atlas skull warped to each patient’s MRI; cortical surface extraction and tessellation into 1 cm^2^ equivalent dipole patches; inverse source estimation with either minimum norm or cortical surface Laplacian constraints; and spectral coherence computed among equivalent dipoles aggregated within Brodmann areas with 1 Hz resolution from 1 to 70 Hz. These analyses revealed characteristic source coherence patterns in each patient during the pre-spike, spike, and post-spike intervals. For one patient with both spikes and seizure onset localized to a single temporal lobe, we observed a cluster of apparently abnormal coherences over the involved temporal lobe. For the other patients, there were apparently characteristic coherence patterns associated with the discharges, and in some cases these appeared to reflect abnormal temporal lobe synchronization, but the coherence patterns for these patients were not easily related to an unequivocal epileptogenic zone. In contrast, simple localization of the site of onset of the spike discharge, and/or the site of onset of the seizure, with non-invasive 256 dEEG was useful in predicting the characteristic site of seizure onset for those cases that were verified by intracranial EEG and/or by surgical outcome.

## 1 Introduction

There is increasing evidence in the recent literature that functional networks of the human brain can be identified through correlation analysis of fluctuations in hemodynamic functional magnetic resonance imaging (fMRI) measures (Fox et al., 2005; Fair et al., 2007, 2008; Van Dijk et al., 2010). To capture the millisecond dynamics of the electrophysiological abnormalities of epilepsy, it is important to apply a similar approach to electrophysiological network analysis of epileptiform discharges, including both spikes and seizures (Gotman et al., 2006; Hamandi et al., 2006; Laufs et al., 2006; Gotman, 2008). Seizures may originate in a focal site of pathological tissue, such as a malformation or lesion of the cortex, but the clinically significant seizure typically involves propagation through some functional networks of the brain.

In a recent study from our laboratory (Ramon et al., 2008; Ramon and Holmes, 2012), non-linear dynamic measures of local correlation among the 256 dense array electroencephalography (dEEG) channels showed abnormally high levels of synchronization over cortical regions that later prove to be seizure onset zones. Because these analyses were conducted with the head surface (scalp) dEEG, they are less precise than synchronization analysis performed with cortical source analysis, assuming, of course, that the cortical source analysis is indeed accurate. In the present study, we developed computational models of the head geometry and conductivity for each patient, including extraction of the cortical surface and tessellation with oriented source dipoles, in order to improve the electrical source analysis of the dEEG measures of epileptiform events. The goal was to apply the synchronization analysis (spectral coherence) to the waveforms of the cortical sources directly.

## 2 Materials and Methods

**Figure 1:**
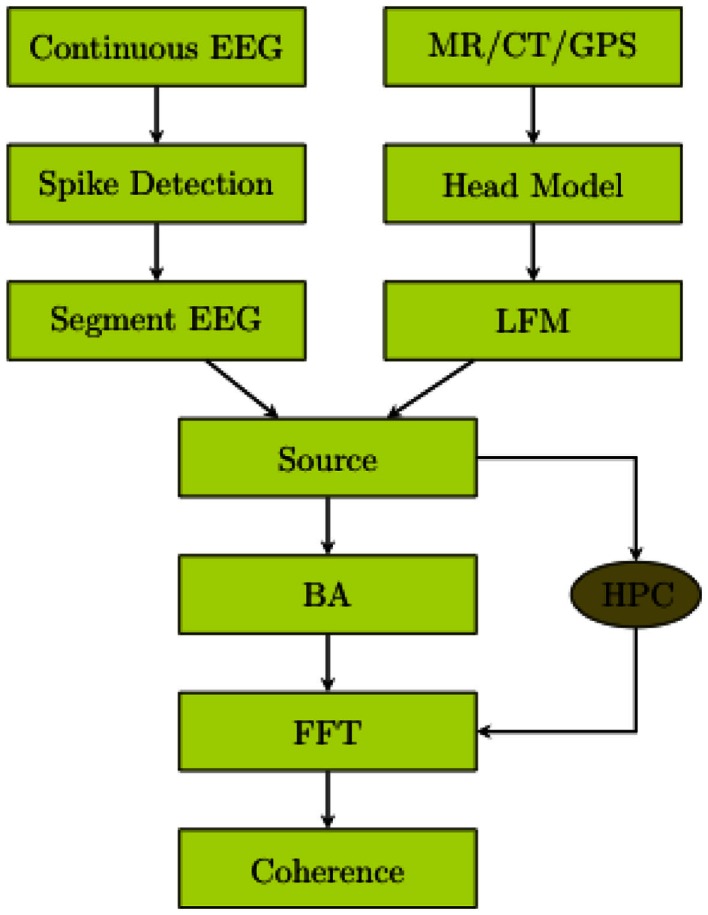
**EEG was continuously recorded**. Spikes were detected. The continuous EEG was segmented. The head volume (MR/CT) was segmented and registered to the electrodes. The lead field matrix was calculated. The cortical source waveforms were estimated. The source waveforms were averaged over Brodmann Areas. The BA waveforms were transformed to the frequency domain by fast Fourier transformation. The coherences of the BA waveforms were computed. If the high performance computing (HPC) power is available, the step of averaging over BAs is not required.

The workflow of the data analysis procedure employed for the present study is presented in Figure 1.

### 2.1 Patients and clinical setting

Patients in this study are five individuals with medically refractory epilepsy who were referred to the University of Washington Regional Epilepsy Center for evaluation and treatment. All were considered as potential candidates for epilepsy surgery. At the time of the evaluation, the subjects ranged in age from 11 to 28 years (mean age = 21 years). Four of the five were males. Duration of epilepsy at the time of the assessment varied from 2 to 16 years (mean = 7 years). Each individual had failed trials of least four standard antiseizure medications, either as monotherapy or in combination, at the time of the presurgical evaluation (range 4–7 drugs).

All patients underwent as part of the presurgical assessment a comprehensive history and clinical examination, routine awake-sleep electroencephalogram (EEG), high-resolution magnetic resonance imaging (MRI), formal neuropsychological testing, and standard EEG-video monitoring. In cases 1, 3, and 4, after a thorough review of these initial studies, the consensus of opinion rendered by epileptologists at Epilepsy Center was that intracranial, subdural strip, or grid electrode EEG-video monitoring was indicated, as the initial non-invasive evaluation failed to provide adequate information regarding seizure onsets. For case 2, intracranial recording was not done because the parents decided against surgery. For case 5, the evidence for temporal lobe onset was sufficiently clear from non-invasive recording to proceed to temporal lobe resection. Prior to invasive monitoring, all subjects underwent 256 dEEG video monitoring to record electrographic activity with a greater degree of spatial resolution and enhance the non-invasive estimate of ictal onsets.

### 2.2 Interictal spike identification

Dense array EEG data were collected during long term monitoring on the epilepsy unit. Identification of spikes in the continuous EEG file was first performed automatically using Persyst (Persyst, San Diego, CA, USA). Briefly, Persyst employs a neural network technique to identify spikes and a statistical clustering algorithm to group spikes according to spatial similarity. The identified spikes were then reviewed by an epileptologist to confirm that identified spikes were indeed spikes and that their grouping were appropriate (i.e., that spikes were topographically homogeneous within each group).

Persyst identified multiple spike types for all patients. However, based on review by the epileptologist, in three patients, only one spike type was confirmed. In the other two patients, the epileptologist confirmed two spike types (see Table 1). For these two patients, we analyzed the data for from both groups but only report results from the analysis of the spike type consistent with their icEEG or resected zone (see Discussion). Once identified in the continuous record, the EEG was segmented centered on the spike peak, with 1.5 s before and after the spike for further analysis (see below).

**Table 1:** **Clinical features of the 5 patients**.

Patient	Gender	Age	10–20 EEG	Spike types (dEEG)	No. spikes	icEEG	Surgery	Outcome
1	Male	27	R temp	L fron^*^	6	R temp	R temp	Engel I
				L occi	3	
2	Male	13	Right	R fron^*^	15	None	None	
3	Male	11	L fron mid	L fron^*^	76	L fron	L fron	Engel II
4	Male	28	Midline	R infe^*^	12	Midline	None	
				L infe	12	
5	Female	19	R fron temp	R infe^*^	56	None	R temp	Engel I

L, left; R, right; *, typical spike type; temp, temporal; fron, frontal; mid, midline; occi, occipital; infe, inferior.

### 2.3 Requirements for electrical source analysis

The understanding of the neural sources of the EEG has been improved in recent years by a number of factors. A first step has been more adequate spatial sampling of the potentials at the head surface with dense sensor arrays (Tucker, 1993). The estimation of the cortical sources of the surface EEG requires accurate characterization of tissue geometries, the EEG sensors positions relative to the tissues, and electrical conductivity of the tissues. Together, the combined information makes up the *electrical head model*.

To create the electrical head model, the EEG sensors must first be located precisely in relation to the head surface. Derivation of sensor positions can be accomplished by using photogrammetric techniques (Russell et al., 2005). Next, the cortical sources must be characterized in the electrical model, typically as electrical dipoles. If the orientation of each patch of cortex is known, such as from cortical surface extraction from the MRI (Dale and Sereno, 1993), then oriented sources may be used. Otherwise, xyz “triple” dipole models must be used for each cortical source, with a considerable loss of constraint, and thus loss of precision for the inverse estimation. Next, a mathematical model of the volume conduction of cortical sources to the head surface is constructed (Malmivuo et al., 1997). When a high-resolution, volumetric MRI can be segmented accurately, a finite difference (or finite element) model can be constructed, specifying the conductivity of each voxel of brain, cerebral spinal fluid, skull, and scalp (Salman et al., 2005a; Turovets et al., 2006). Spherical or boundary element models cannot account for the complexity of the conductive compartments, particularly for inferior head regions that are critical for the propagation of discharges basal brain structures (basal temporal and orbital frontal) to the head surface (including the face and neck). Because the resistive skull must be specified precisely, fitting an volumetric x-ray computed tomography (CT) image to the head model is a critical step (Salman et al., 2005b).

Finally, given the complex *forward model* specified by these facts of tissue geometry and electrical conductivity, the estimation of cortical source activity is made through an *inverse model*. When the parameters of the electrical head model are specified loosely or inaccurately, such as with electrodes only assumed to be at typical positions, the cortical sources at unknown orientations and therefore modeled as dipole “xyz” triples, and the head conductivity modeled loosely as concentric spheres, the constraints on the source estimation are so loose as to create large uncertainty bounds. Fundamentally, the inverse model is *ill-posed*, and source estimation is an approximation in the best case. The ill-posed inverse model may be stabilized through regularizing with various methods, including the minimum norm (Ou et al., 2008), 3D Laplacian smoothing (Pascual-Marqui et al., 1994), statistical standardization of the projection of each source to the sensors (Pascual-Marqui, 2002), ECD (equivalent current dipole) (Fischer et al., 2005), or beamforming (Gross et al., 2001). In previous research on localization of spike and seizure onset, reasonable results have been created with dEEG (128 and 256 channel) measurement, using a realistic head conductivity atlas (finite difference model from an atlas MRI) with cortical triples and various methods of regularization and smoothing of the inverse (Michel et al., 2004; Tucker et al., 2007, 2009; Guggisberg et al., 2008; Brodbeck et al., 2010; Holmes et al., 2010a,b; Holmes, 2011; Bouet et al., 2012; Yamazaki et al., 2012). These results suggest that more accurate solutions for the inverse problem can be achieved with a more accurate volume conductor head model.

In the present study, we improved the accuracy of the electrical head model for each patient by specifying cortical source dipoles whose orientation was known from extracting the gyral-sulcal cortical surface for each patient. The goal was to develop an accurate source analysis for major regions of the cortex (Brodmann areas) that could then support analysis of electrophysiological coherence that may be related to the pathological activity in cerebral networks engaged by the patient’s epileptic discharges.

### 2.4 Constructing the electrical head model

The electrical head model was constructed with the BrainK software (Li et al., 2006). For each patient and each EEG recording session, sensor positions of the 256-channel Geodesic Sensor Net were determined with multi-camera geodesic photogrammetry system (GPS) (Russell et al., 2005). The data were then registered with the patient’s head model, derived from structural MRIs. The patient’s volumetric MRI was segmented into scalp, skull, CSF, and brain gray and white matter. Accurate segmentation was optimized with a unique *relative thresholding* algorithm, which corrects for the inhomogeneity of MR images. Characterization of the detailed geometry of the skull was accomplished through non-linear warping of an atlas skull CT to the patient’s MRI image. Analysis of the electrical source activity within the patient’s MRI allowed inspection of the convergence of anatomical features with the epileptic discharges. The MRI for Patient 3, for example, showed a blurred gray-white boundary in the left dorsal frontal region, possibly suggesting a malformation, and this structural feature proved to be co-located with the patient’s typical source-localized epileptiform discharges.

With the cortical ribbon segmented by its inner and outer table, the outer (gray to CSF) cortical surface was meshed, and then cast into a geodesic graph theoretical representation with the Chaco graph algorithms (Sandia National Laboratories). The graph representation allows flexible tessellation of the cortical surface, and flexible representation of mathematical constraints defined on that surface, such as the Cortical Surface Laplacian (CSL) defined below. For the present analysis, 1 cm^2^ cortical patches (about 1200 per hemisphere) were created. For each patch, the average orientation within that patch was characterized by computing the surface normal for each triangle in that patch, then computing the vector sum across all patch triangles as the representation of the equivalent dipole orientation for that patch.

### 2.5 Inverse method

The use of oriented dipoles on the cortical surface provides an important constraint on the inverse source estimation process that appears to improve accuracy considerably. Much of the ambiguity in the relation of cortical electrical activity to the head surface measurements is created by the extensive gyral and sulcal folding of the individual patient’s cortex. The requirement, of course, is that the position of the EEG sensors in relation to the oriented sources must be specified precisely; otherwise the misalignment will result in incorrect source attributions.

Given accurate geometric alignment, accuracy in the conductivity of the volume conduction model is then required, including the precise geometry of the skull, such as from CT. Given these several measurement constraints on the forward model, we have observed that minimal regularization constraints on the inverse estimation, such as the minimum norm, yield accurate results. Support for this accuracy has come from initial (unpublished) validation studies with dEEG mapping of individual sensory and motor potentials, with similar results as seen with fMRI for that individual. These conclusions on the decreased importance of regularization are consistent with previous reports using oriented cortical sources (Dale and Sereno, 1993; Ou et al., 2008; Knosche et al., 2013).

Although reasonably accurate with the orientation constraints, the minimum norm remains a non-specific constraint, and is not an optimal fit to the physiological properties of electrophysiological activity of the cortex. To optimize the inverse constraints that are appropriate to our knowledge of localized cortical activity, we have developed an inverse regularization or smoothing constraint that makes use of the knowledge of adjacency of dipoles on the extracted 2-dimensional cortical surface. This is the *Cortical Surface Laplacian*, computed as the Laplacian operator for a given dipole source against the (typically 6) neighboring dipoles on the 2D cortical surface. There are both physical and physiological rationales for this constraint. Physically, only synchronous activity of adjacent, laminar cortical neurons (thought to be pyramidal cells and their apical dendrites) is capable of generating the far field observed by head surface electrodes (Nunez and Srinivasan, 2005). Physiologically, the columnar and hyper-columnar organization of the human cortex (Jones, 2009) makes it plausible that synchronous activation – perhaps even as large as a 1-cm^2^ patch – might be reasonable.

The CSL constraint is then used to smooth or regularize the inverse estimation, to avoid inappropriate mathematical results that are possible with the instabilities of the matrix inverse. The inverse estimation then: (1) minimizes the difference between the forward oriented cortical source model and the observed surface potentials (the *data fidelity* *term*) and (2) minimizes the CSL as the *regularization term*. With Φ as the measured surface potentials, *K* as the lead field or forward volume conduction matrix, *J* as the cortical sources, and ε as the error term, the surface potentials can be modeled as the projection of the cortical source activity through the head volume model, plus error:

Φ=KJ+ε.

The inverse formulation minimizes the data fidelity term plus the regularization term:

$$Ĵ=\arg\underset{J}{\min}\left\{{∥\Phi -KJ∥}^{2}+\alpha {∥WJ∥}^{2}\right\},$$

where *W* comprises a discrete spatial Laplacian operator defined by

$$w_{ij}=\left\{\;\begin{array}{cc} -1 & \text{if }i\text{ = }j\\ \frac{1}{N_{i}} & \text{if }i\;\neq \text{ j and voxel }j\text{ is a neighbor of voxel }i\\ 0 & \text{otherwise} \end{array}\right.,$$

where *N_i_* is the number of cortical surface dipole neighbors (patches or voxels) to dipole *i*.

### 2.6 Investigating pathologies of cortical coherence

The goal of the present study was to implement and then evaluate these several advances in individual head modeling for dEEG source waveforms to investigate cortical network patterns (source coherence) in relation to epileptiform discharges (spikes) in patients being examined for possible neurosurgical resection of the epileptic focus. The hypothesis was that abnormal patterns of cortical source coherence during interictal events may reveal abnormal patterns of electrophysiological synchronization associated with the seizure onset zone. For each of the five patients, we selected characteristic interictal spikes and then clustered these to insure that all spikes in the cluster had a common head surface topography in the 256 surface dEEG array. The cortical source waveforms were computed with the patient’s individual head model with the CSL constraint for three intervals: 1 s before the spike; 1 s centered on the spike, and 1 s after the spike.

Coherence is the standardized cross-spectral density computed between two signals, *x* and *y*:

$$\begin{array}{llll} & X,Y:\text{Fast Fourier Transform of }x\text{ and }y & & \\ & S_{XX}\left(f\right):\text{autospectrum of }X\text{at frequency }f & & \\ & S_{YY}\left(f\right):\text{autospectrum of }Y\text{at frequency }f & & \\ & S_{XY}\left(f\right):\text{cross - spectrum of }X\text{ and }Y\text{at frequency }f & & \\ & Coherence\left(f\right)=\frac{S_{XY}\left(f\right)}{\sqrt{S_{XX}\left(f\right)\cdot S_{YY}\left(f\right)}}. & & \end{array}$$

Just as the power spectrum describes frequency spectrum of the variance of one signal, the cross-spectrum describes the frequency spectrum of the covariance between two signals. Because the covariance is affected by the amplitude of the component signals, the coherence measure is standardized, by dividing the cross-spectrum by the square root of the product of the power spectra. Coherence is thus the frequency domain analog of the Pearson correlation coefficient (which is covariance of two variables divided by the square root of the product of their respective variances).

With the 1-s epoch for each interval (pre-spike, spike, and post-spike), the frequency resolution was 1 Hz, and coherences for 1–70 Hz were examined. The frequency range was divided into five frequency bands; delta, theta, alpha, beta, and gamma. With ∼2400 patches of size 1 cm^2^ for the cortical surface, and thus 2400 equivalent source dipoles, there are 2,878,800 comparisons [(*N*^2^ − *N*)*/*2]. With 5 coherence values for each comparison, the coherence matrix for each 1-s interval becomes somewhat large (∼360 million entries). For this preliminary study, we therefore grouped the dipoles within the BAs for each hemisphere, and averaged all source waveforms in each BA. For the grouping, the individual’s brain was transformed to Talairach space, and the BAs for each cortical patch were determined with the Talairach Daemon (Lancaster et al., 2000). This resulted in about 40 BAs per hemisphere (depending on the anatomy of the patient’s cortex and its alignment with the Talairach transform). This resulted in ∼3160 cross-signal coherences in each matrix. For an overview visualization of the coherence matrices, each was plotted as an 80 × 80 matrix, with the coherence entries scaled by a color palette (Figure 3). Each matrix shows coherence values in the form of a color palette, with green to blue as lower coherence and yellow to red as higher coherence. The variables on the axes are the approximate Brodmann areas (slightly different areas are reflected by these numbers for each patient, depending on the patient’s cortical anatomy and alignment with the Talairach atlas). In each matrix, the intra-hemispheric coherences for the right hemisphere are in the upper left, and intra-hemispheric coherences for the left hemisphere in the lower right, and inter-hemisphere coherences in the lower left and upper right quadrants. In general, for all patients, intra-hemispheric coherences are higher than inter-hemispheric coherences. For a more anatomical visualization, the cortical patch dipoles were plotted in their 3D positions in a glass brain (the vertical or axial projection was made transparent), and the highest 100 coherences from each interval were represented by a line between the coherent signals (e.g., Figure 4).

## 3 Results

For each patient, we first summarize the standard clinical evaluation: (1) non-invasive spike and seizure localization with conventional (Ten-Twenty System) EEG; (2) non-invasive spike and seizure localization with 256 dEEG; (3) icEEG when that was done; (4) the decision whether the patient was a surgical candidate; and (5) if so, the surgical outcome (Engel class) at the present time in Table 1. Next, the coherence analyses for the interictal events (spikes) are presented, for the pre-spike, spike, and post-spike intervals. One typical spike cluster (comprising spikes with similar head surface topographies) is described for each patient; the set of spike clusters obtained for that patient and differences in coherence patterns for the other clusters is summarized in each case. The peak coherences are shown for all 3 intervals (pre-spike, spike, and post-spike). In each case, the differences across differing spike clusters for that patient, and the differences between pre-spike, spike, and post-spike intervals are summarized.

### 3.1 Patient 1

EEG: right temporal seizures, not well localized on the scalp.
dEEG: right medial temporal localization for both spikes and seizures.
icEEG: right medial temporal localization for both spikes and seizures.
Surgery: right temporal resection.
Outcome: seizure free (Engel Class I) for 1 year.
Coherence analysis: cortical patch tessellation resulted in 2240 patch dipoles for Patient 1 which were classified into 82 Brodmann areas (41 per hemisphere). For a typical cluster of left-frontal spikes (*N* = 6), the coherence matrices are shown in Figure 3.

**Figure 2:**
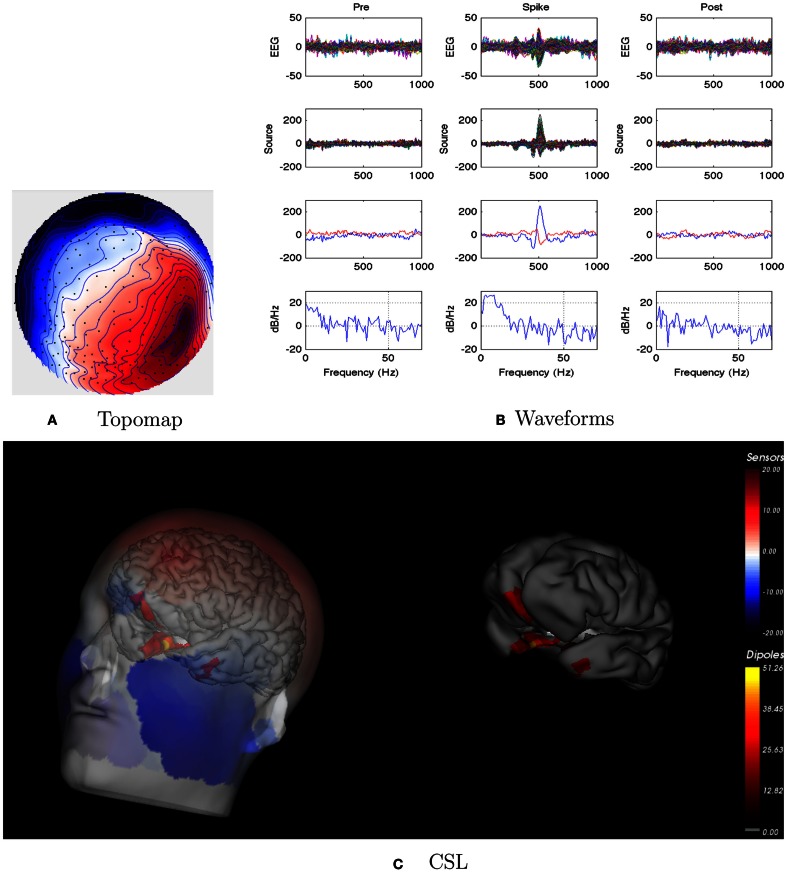
**Patient 1’s non-invasive dEEG localization of a typical (left-frontal) spike**. **(A)** Topographic map of scalp potentials at peak of spike (which is used for spike classification). Orientation is top looking down with the nose at the top; red = positive, blue = negative, white = zero. The right inferior (face and neck) spatial gradient (described by the isopotential lines) suggests a right anterior temporal source, even though the spike (i.e., negative features) was located at left-frontal recording sites. **(B)** Waveforms of EEG from all channels (top row), source waveforms from all 2240 sources (second row), source waveform from dipole that shows maximal activity at spike peak (third row, blue trace) and source waveform from dipole that shows maximal activity at 50% of spike peak (third row magenta trace), and the frequency spectra of the blue source waveforms (third row) shown in forth row. Note that the dipole location of the blue trace is in the left temporal lobe and the magenta trace in the third row is in the right anterior temporal lobe, consistent with the icEEG data and resection zone. In **(B)**, each column represents 1 s of data from a 3-s segment. Middle column represent data centered on the spike peak. **(C)** Electrical source localization with the patient’s individual electrical head model. Left: scalp potential reconstructed from the source estimate (at 50% of spike peak). Right: partially inflated brain illustrating the dominant source associated with the data illustrated in left figure (location of dipole described by magenta trace in **(B)**, third row). Data was thresholded to only show top 5% activity.

**Figure 3:**
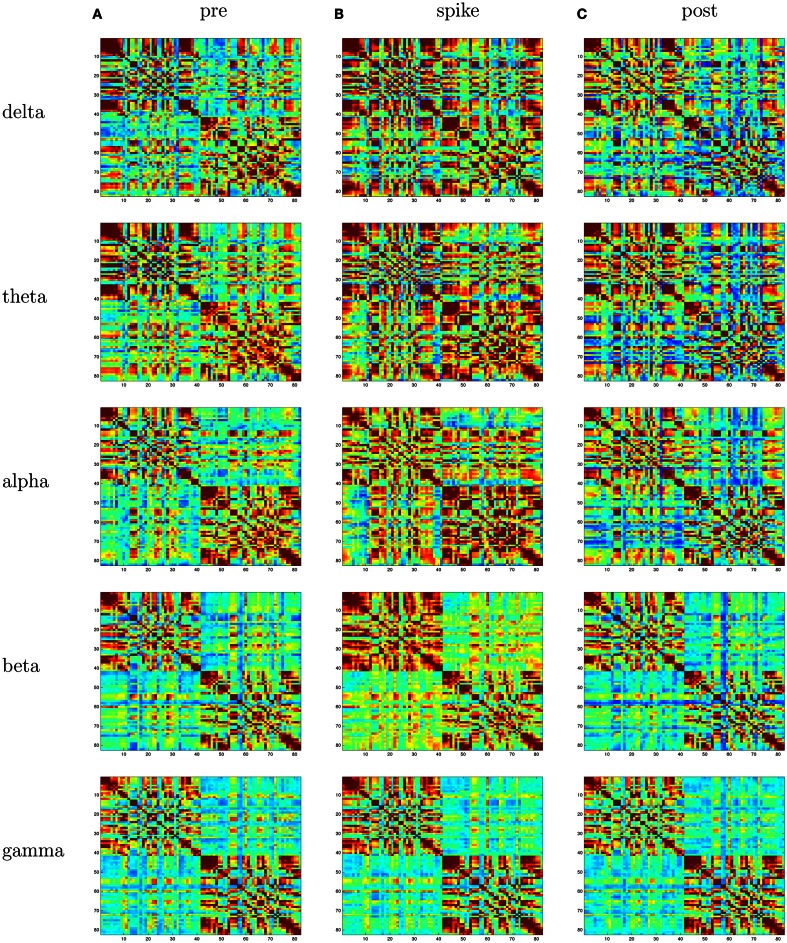
**Patient 1’s dEEG cortical source coherence matrices for the delta (0–4 Hz), theta (4–8 Hz), alpha (8–13 Hz), beta (13–30 Hz), and gamma (30–70 Hz) frequency bands, computed between 82 Brodmann areas (the numbers identify slightly different BAs for each patient, depending on alignment)**. These coherences are computed for the 1-s pre-spike **(A)**, the 1-s centered on the spike **(B)**, and the 1-s post-spike **(C)** intervals.

**Figure 4:**
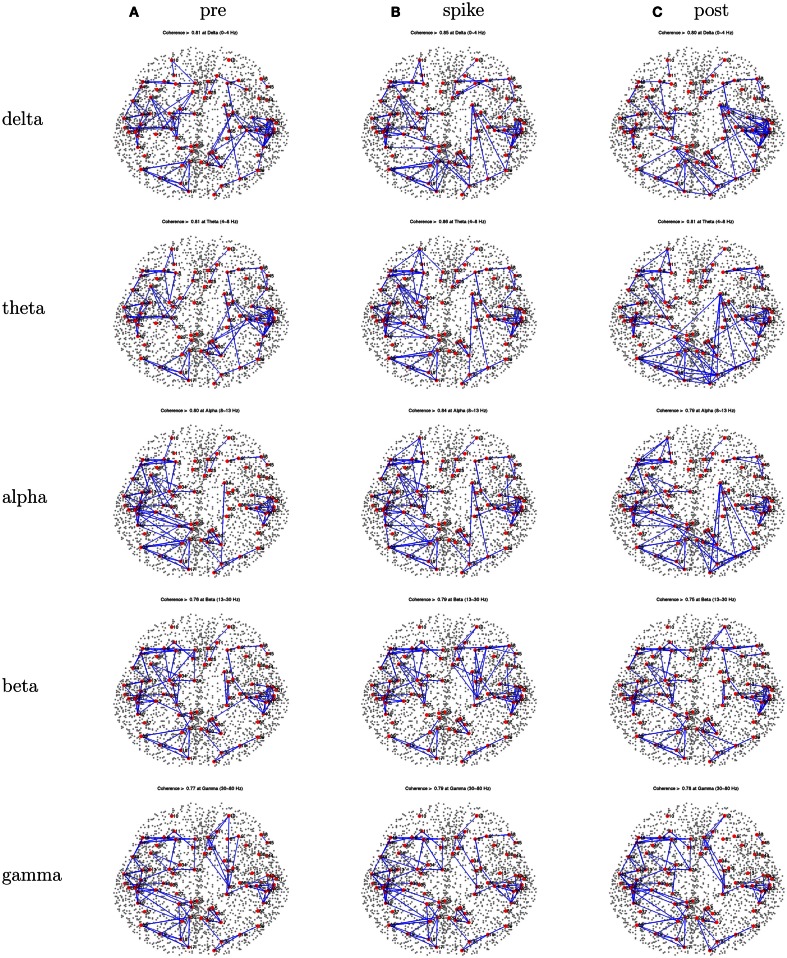
**Patient 1’s dEEG cortical source coherence peaks (highest 100 of ∼6400) for pre-spike (A), spike (B), and post-spike (C) intervals of the five frequency bands**.

For this patient, the spike clustering show a group with a left-frontal distribution. However, analysis of the spike at 50% maximum amplitude (as recommended in previous work by Lantz et al., 2003) show a dominant right-lateralized source in the temporal region (see Figure 2). An overview summary of Patient 1’s spike coherences is given in Figure 3. The pre-spike interval coherences appear unremarkable, with the possible exception of certain low inter-hemispheric values (blue) for the gamma band. The spike-interval coherences show higher values overall, including in the inter-hemispheric quadrants for the lower (delta, theta, alpha) bands. The post-spike interval coherences return to somewhat lower values, with perhaps a specific decrease in the inter-hemispheric values for the lower (delta, theta, alpha) bands. The peak coherences for this same spike cluster for Patient 1 are shown in Figure 4. The patterns for peak coherences for Patient 1 are similar for pre-spike, spike, and post-spike intervals, with a strong delta cluster (and perhaps theta) over the right temporal region. There seems to be a similar pattern across bands in which there are fairly distributed peak coherences across the left hemisphere, but a focal cluster in the temporal area for the right hemisphere. Somewhat higher values of the distributed left hemisphere coherences are observed for the alpha band in the pre-spike interval. The right temporal cluster may be particularly strong for the delta band in the spike interval. The post-spike interval includes what appears to be stronger occipital coherences, including inter-hemispheric, particularly for the theta band.

### 3.2 Patient 2

**Figure 5:**
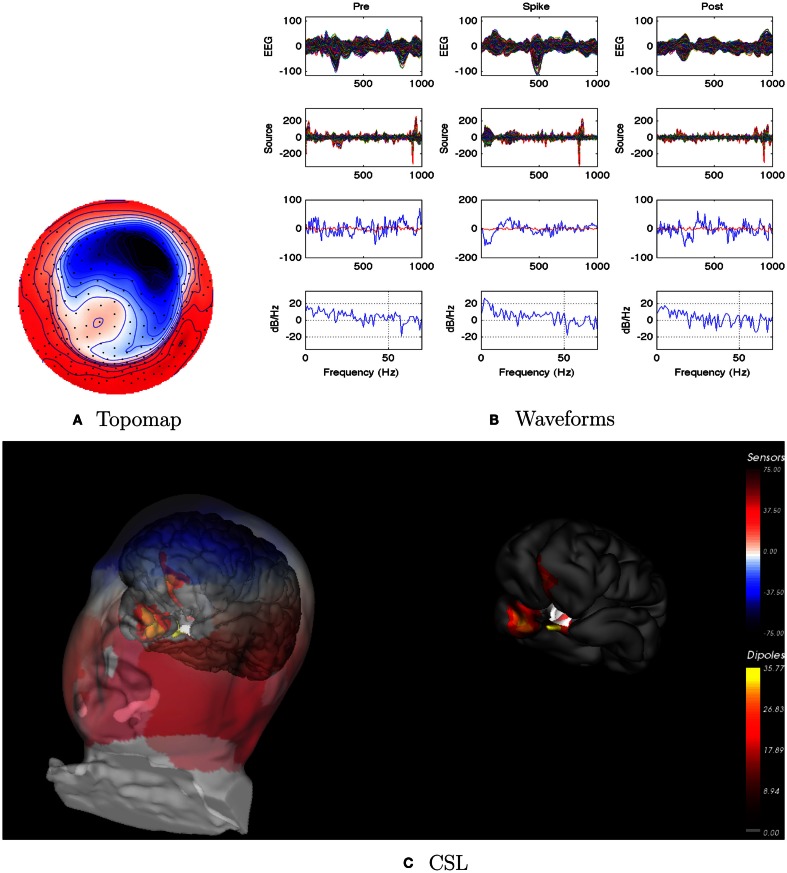
**Patient 2’s non-invasive dEEG localization of a typical (right frontal) spike**. **(A)** Topographic map of scalp potentials at peak of spike (which is used for spike classification). Orientation is top looking down with the nose at the top; red = positive, blue = negative, white = zero. **(B)** Waveforms of EEG from all channels (top row), source waveforms from all 2272 sources (second row), source waveform from dipole that shows maximal activity at 50% of spike peak (third row, blue trace) and source waveform from dipole at a distance from maximal location (third row magenta trace), and the frequency spectra of the blue source waveforms (third row) shown in forth row. Note that the dipole location of the blue trace is in the right temporal lobe. In **(B)**, each column represents 1 s of data from a 3-s segment. Middle column represent data centered on the spike peak. **(C)** Electrical source localization with the patient’s individual electrical head model. Left: scalp potential reconstructed from the source estimate (at 50% of spike peak). Right: partially inflated brain illustrating the dominant source associated with the data illustrated in left figure [location of dipole described by blue trace in **(B)**, third row]. Data was thresholded to only show top 5% activity.

EEG: interictal events (spikes) were right sided. Seizures seemed to be on the right, but the pattern was unclear.
dEEG: right temporal localization for both spikes and seizure onset in Figure 5.
icEEG: not done.
Surgery: none. Parents decided not to pursue surgical resection.
Coherence analysis: cortical patch tessellation resulted in 2272 patch dipoles for Patient 2 for 83 Brodmann Areas. A cluster of 15 right frontal spikes was identified. In the dEEG analysis (both atlas and individual cortical source localization), the onset of these spikes was localized to the right temporal area, and there was rapid propagation to sources in the right frontal area (creating the right frontal topography for the spike peak).

**Figure 6:**
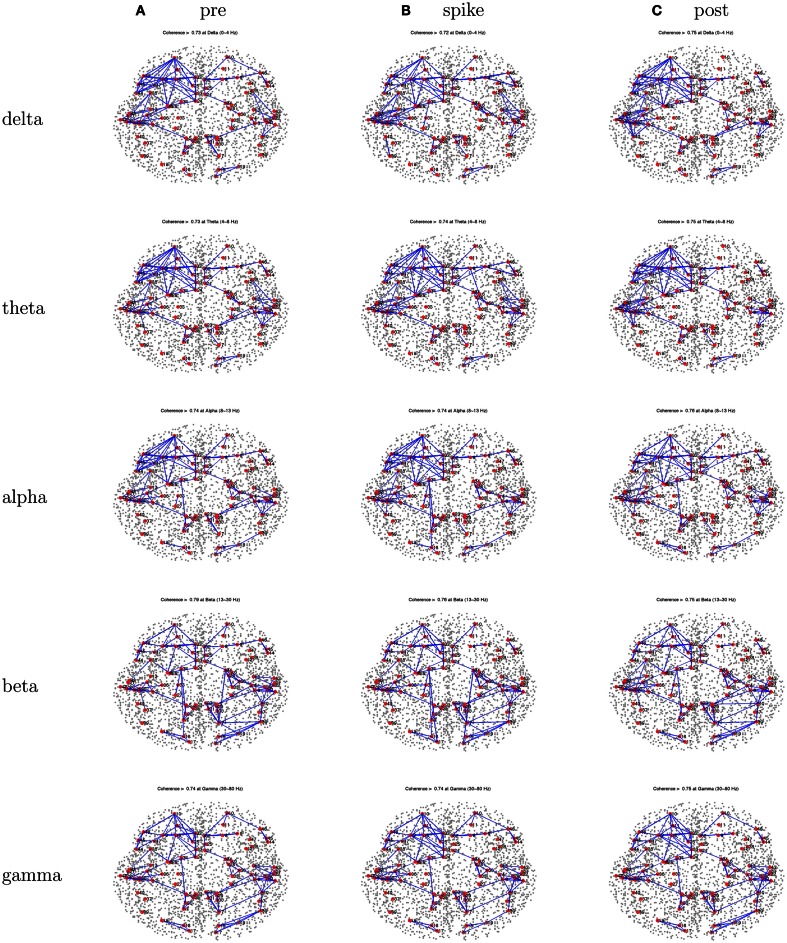
**Patient 2’s dEEG cortical source coherence peaks (highest 100 of ∼6400) for pre-spike (A), spike **(B)**, and post-spike **(C)** intervals of the five frequency bands**.

Patient 2’s peak coherences were generally similar for the pre-spike, spike, and post-spike intervals, as was observed for all patients in this series (Figure 6). For the delta, theta, and alpha bands particularly, there was a cluster of peak coherence over the left-frontal and temporal regions. For beta and gamma bands, there was a cluster of peak coherence over the right posterior regions.

### 3.3 Patient 3

**Figure 7:**
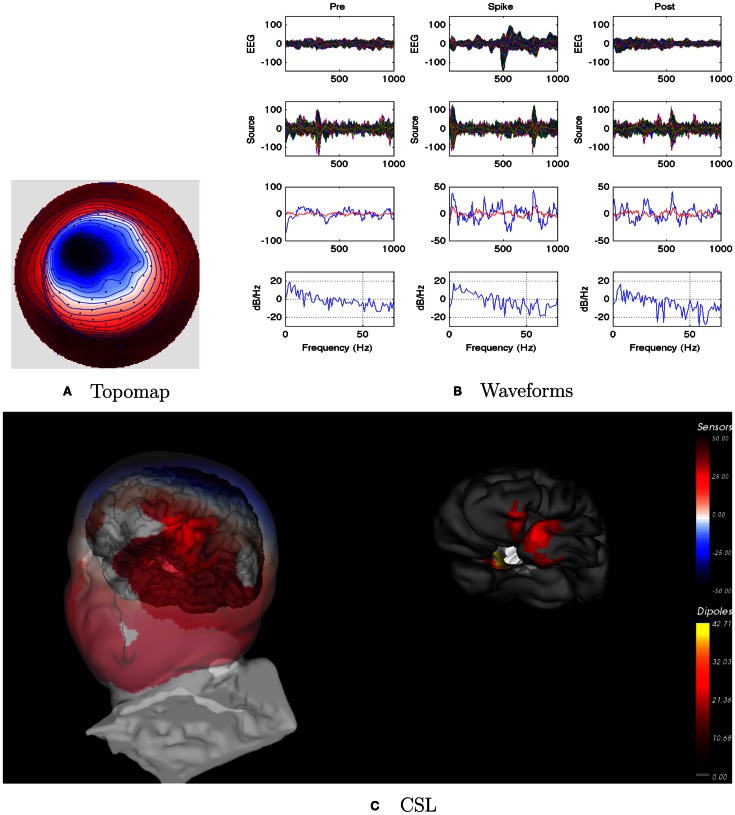
**Patient 3’s non-invasive dEEG localization of a typical (left-frontal) spike**. **(A)** Topographic map of scalp potentials at peak of spike (which is used for spike classification). Orientation is top looking down with the nose at the top; red = positive, blue = negative, white = zero. **(B)** Waveforms of EEG from all channels (top row), source waveforms from all 2260 sources (second row), source waveform from dipole that shows maximal activity at 50% of spike peak (third row, blue trace) and source waveform from dipole at a distance from maximal location (third row magenta trace), and the frequency spectra of the blue source waveforms (third row) shown in forth row. Note that the dipole location of the blue trace is in the left-frontal lobe. In **(B)**, each column represents 1 s of data from a 3-s segment. Middle column represent data centered on the spike peak. **(C)** Electrical source localization with the patient’s individual electrical head model. Left: scalp potential reconstructed from the source estimate (at 50% of spike peak). Right: partially inflated brain illustrating the dominant source associated with the data illustrated in left figure (location of dipole described by blue trace in **(B)**, third row). Data was thresholded to only show top 5% activity.

EEG: seizures observed with apparent onset in the left-frontal midline.
dEEG: similar left-frontal midline localization for both spikes and early seizure activity, but evidence of early onset for both spikes and seizures in left temporal region in Figure 7.
icEEG: seizure activity observed over left-frontal midline cortex.
Surgery: resection of left-frontal midline.
Outcome: after 1 year, seizures are improved (Engel Class II).
Coherence analysis: cortical patch tessellation resulted in 2260 patch dipoles for Patient 3 for 80 Brodmann Areas. Spike clustering showed the predominance of spikes showed a left-frontal topography (*N* = 76); these were examined with coherence analysis.

**Figure 8:**
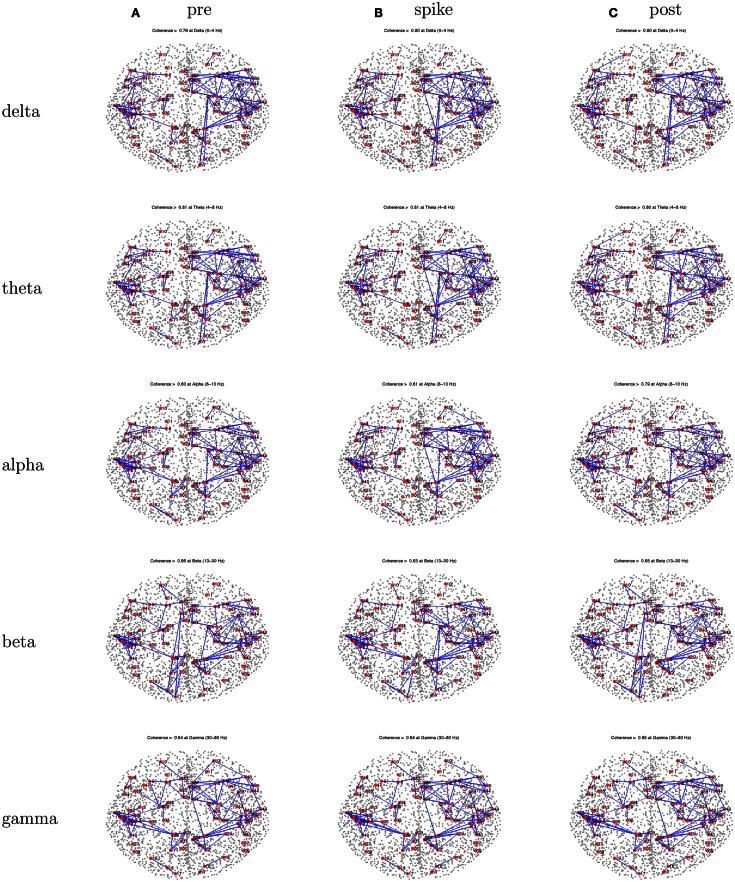
**Patient 3’s dEEG cortical source coherence peaks (highest 100 of ∼6400) for pre-spike (A), spike (B), and post-spike (C) intervals of the five frequency bands**.

The peak coherence analysis for Patient 3 (Figure 8) showed fairly similar patterns across the three intervals. Coherences are somewhat higher for right hemisphere cortical sources than left hemisphere cortical sources. Somewhat higher coherences are seen for the beta band than for other frequencies, and for the gamma band for the right hemisphere intra-hemispheric values particularly. The pattern seems like a mirror image of that for Patient 1, with a distributed frontotemporal cluster of peak coherences over the right hemisphere, but a more focal pattern of peak coherences over the left temporal region.

### 3.4 Patient 4

**Figure 9:**
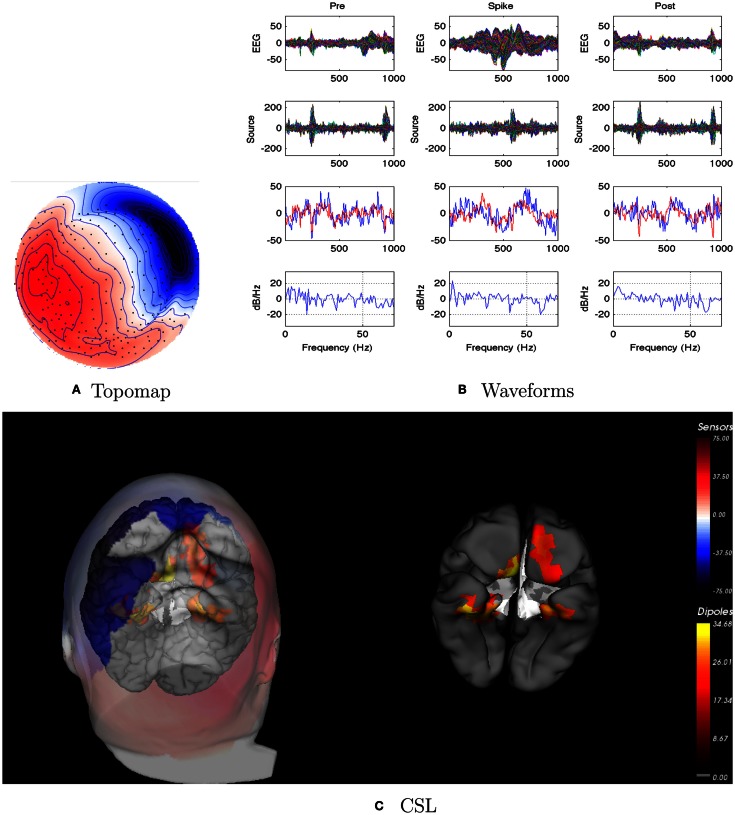
**Patient 4’s non-invasive dEEG localization of a typical (right inferior) spike**. **(A)** Topographic map of scalp potentials at peak of spike (which is used for spike classification). Orientation is top looking down with the nose at the top; red = positive, blue = negative, white = zero. **(B)** Waveforms of EEG from all channels (top row), source waveforms from all 2247 sources (second row), source waveform from dipole that shows maximal activity at 50% of spike peak (third row, blue trace) and source waveform from dipole at a distance from maximal location (third row magenta trace), and the frequency spectra of the blue source waveforms (third row) shown in forth row. In **(B)**, each column represents 1 s of data from a 3-s segment. Middle column represent data centered on the spike peak. **(C)** Electrical source localization with the patient’s individual electrical head model. Left: scalp potential reconstructed from the source estimate (at 50% of spike peak). Right: partially inflated brain illustrating the dominant source associated with the data illustrated in left figure (location of dipole described by blue trace in **(B)**, third row). Data was thresholded to only show top 5% activity.

EEG: seizures appeared to involve midline structures.
dEEG: seizures appeared to involve right central and midline structures. Interictal analysis of several spike clusters (right inferior, left inferior) showed the onset of each of these differing spike discharges appeared to be in the left and right medial temporal cortex as well as bilateral mediofrontal cortex (Figure 9).
icEEG: seizures involved both left and right midline cortex.
Surgery: none. Patient was judged not to be a surgery candidate.
Coherence analysis: cortical patch tessellation resulted in 2247 patch dipoles for Patient 4 for 81 Brodmann Areas. Spike clustering showed the three differing peak topographies: midline frontal, left inferior, and right inferior. The right inferior spike cluster was selected for coherence analysis (*N* = 12).

**Figure 10:**
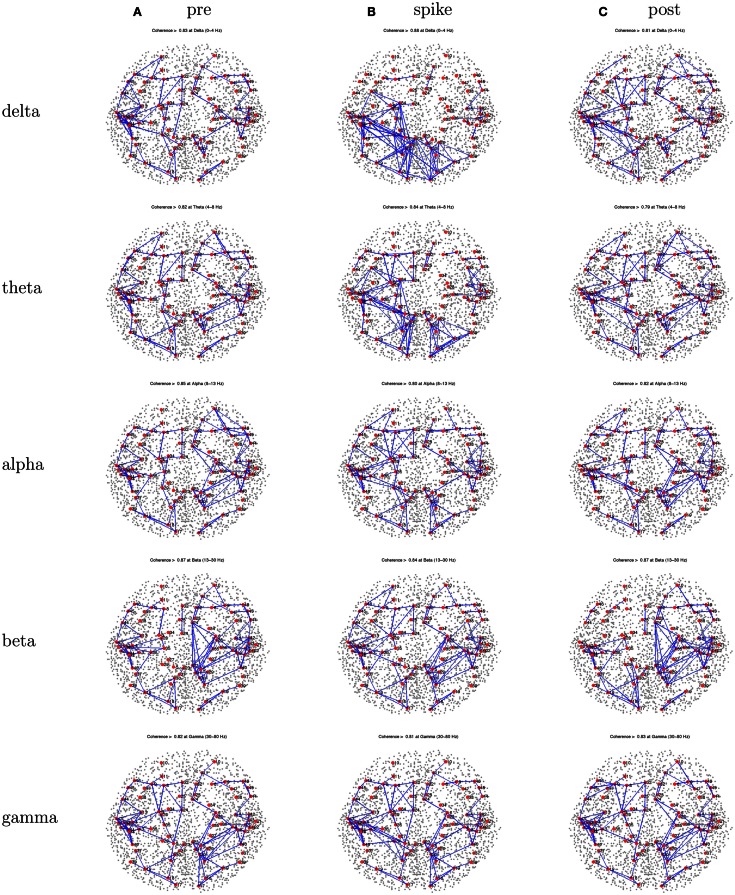
**Patient 4’s dEEG cortical source coherence peaks (highest 100 of ∼6400) for pre-spike (A), spike (B), and post-spike (C) intervals of the five frequency bands**.

The peak coherences of the spike interval for Patient 4 were possibly more focal than for the other intervals, implicating the left inferior region for the delta band (Figure 10). However, as is typical in these analyses, the peak coherence patterns were roughly similar for the three (pre-spike, spike, and post-spike) intervals. For Patient 4, there were suggestions of a tight temporal lobe cluster, in this case on the left, which seemed to engage other left posterior sites for the delta.

### 3.5 Patient 5

**Figure 11:**
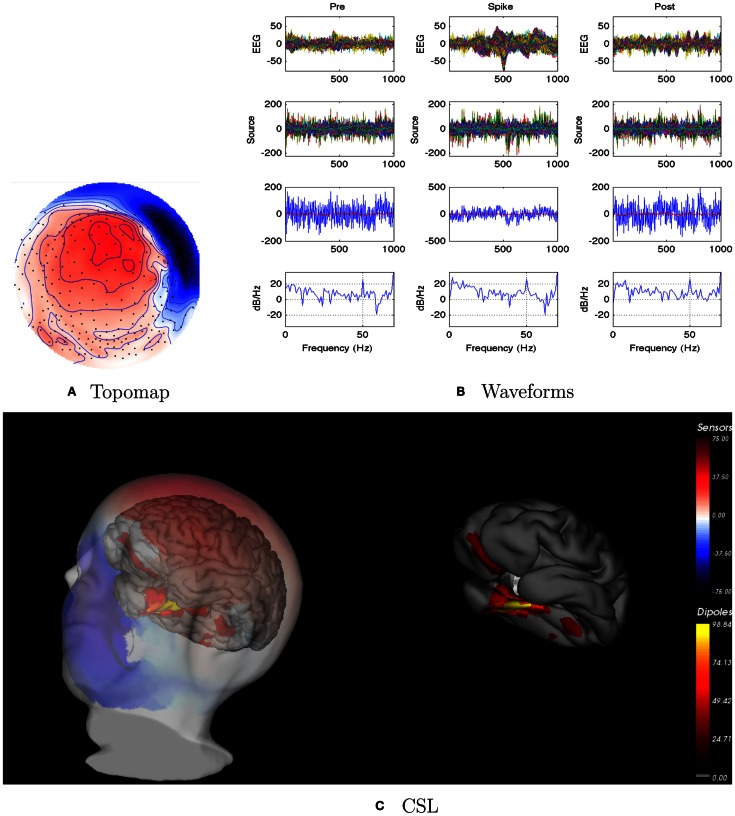
**Patient 5’s non-invasive dEEG localization of a typical (right inferior) spike**. **(A)** Topographic map of scalp potentials at peak of spike (which is used for spike classification). Orientation is top looking down with the nose at the top; red = positive, blue = negative, white = zero. **(B)** Waveforms of EEG from all channels (top row), source waveforms from all 2244 sources (second row), source waveform from dipole that shows maximal activity at 50% of spike peak (third row, blue trace) and source waveform from dipole at a distance from maximal location (third row magenta trace), and the frequency spectra of the blue source waveforms (third row) shown in forth row. In **(B)**, each column represents 1 s of data from a 3-s segment. Middle column represent data centered on the spike peak. **(C)** Electrical source localization with the patient’s individual electrical head model. Left: scalp potential reconstructed from the source estimate (at 50% of spike peak). Right: partially inflated brain illustrating the dominant source associated with the data illustrated in left figure (location of dipole described by blue trace in **(B)**, third row). Data was thresholded to only show top 5% activity.

EEG: seizures appeared to involve right frontotemporal areas, and spikes were right sided.
dEEG: both spikes and seizures showed a right medial temporal lobe onset in Figure 11.
icEEG: not done.
Surgery: right temporal lobe resection. Patient is seizure free (Engel Class I) for about 1 year.
Coherence analysis: cortical patch tessellation resulted in 2244 patch dipoles for Patient 5 for 82 Brodmann Areas. Spike clustering showed only one topography: right inferior spikes (*N* = 56).

**Figure 12:**
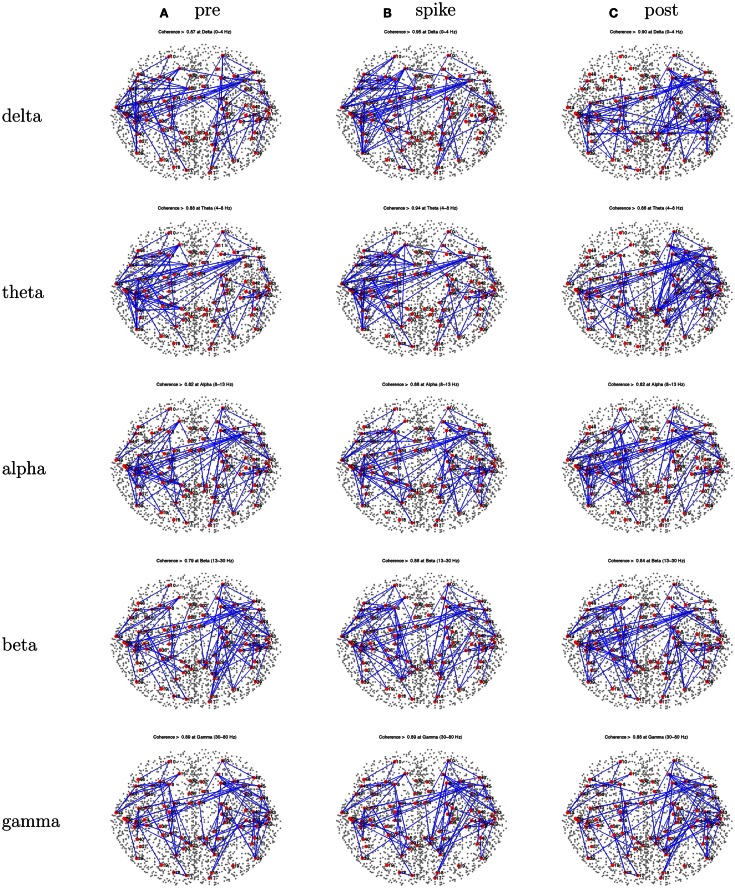
**Patient 5’s dEEG cortical source coherence peaks (highest 100 of ∼6400) for pre-spike (A), spike (B), and post-spike (C) intervals of the five frequency bands**.

Patient 5’s peak coherences (Figure 12) show a unique pattern of left-frontal and temporal coherences, together with high coherences between these regions with a source in the right frontal area. This pattern is observed for the lower frequency bands. Whereas features of this pattern can be seen for the beta and gamma bands, there are increased coherences over right hemisphere regions, particularly for the frontal and temporal regions for the gamma band.

## 4 Discussion

The results of the coherence analyses in the present study must be seen as preliminary. We are only beginning to validate the oriented cortical source analysis constructed from individual head models. The present data are the first we have examined with oriented cortical source analysis of epileptic discharges and the present results are perhaps most useful in illustrating the approach to characterizing cortical network electrophysiology rather than providing definitive evidence on epileptic networks.

In a similar vein, DICS (dynamic imaging of coherent sources) has been used to localize the epileptic spikes using oscillatory features (Guggisberg et al., 2008; Bouet et al., 2012). The DICS method focuses on the source localizations of the spike-related high frequency activity (>20 Hz). The present study considered low as well as high frequencies, using coherence analysis to attempt to discern pathological networks contributing to the epileptic spikes.

In considering the accuracy of the oriented cortical source analysis with the CSL constraint, we observed good correspondence in the present CSL analyses of spike and seizure onset and the atlas-based model (dipole triples with the MNI average cortex and a generic head conductivity model) implemented in Geosource 2.0. Given the previous validation of the atlas (Geosource 2.0) analysis in relation to intracranial recordings (Yamazaki et al., 2012) and surgical outcome (Holmes et al., 2008, 2010a) the present results imply that the CSL inverse is at least roughly correct when applied to the oriented cortical sources constructed for the individual patient.

Given the previous findings of characteristic patterns of EEG synchronization over the seizure onset zone in the scalp (head surface) EEG (Ramon and Holmes, 2012), we organized the present study to examine cortical source coherence, rather than EEG electrode channel coherence. On first principles, we would expect the cortical source coherence – even with the summation over the large Brodmann areas – to reveal more about cortical function than the badly superposed gyral and sulcal fields that are summed at any given surface electrode.

Whether the coherence analysis of the source activity before, during, and after the spike will yield clinically meaningful information remains to be seen. It is noted here that, because this study represent the first systematic study of the approach we employ here, we also analyzed several aspects of the data but do not report findings in detail here. First, for those patients with confirmed multiple spike types (e.g., Patients 1 and 4), the coherence patterns were not substantially different between the spike types at the three time intervals. Second, we also examined random data from spike-free intervals, and the coherence patterns for each patient was similar to their spike-interval coherence patterns. Nevertheless, the overall results do point to interesting possibilities.

Patient 1 did show a promising result. He manifested a single seizure focus in the right temporal lobe that, even though the peak of the spike indicated a left-frontal focus, when resected, resulted in his being seizure free. His characteristic interictal spikes were associated with a tight pattern of right temporal lobe peak coherences that dominated over other coherences in the right hemisphere. Of course, an obvious interest is whether seizure onset is associated with a similar pattern of abnormal synchronization. We chose to focus on interictal events for this preliminary study because the dEEG data quality is high, and there are many spikes that provide for statistical stability of the results. Nonetheless, it is clearly important to extend these methods to analysis of cortical synchronization associated with seizure onset.

Patient 5 also showed spikes and seizures localized to the right temporal lobe, and a right temporal lobectomy eliminated seizures (at least for the 1-year follow up to date). Yet Patient 5 showed a dominance of peak coherences over *left* frontotemporal regions for most frequency bands. It was the case that the gamma band showed the strongest coherences for Patient 5, where a right-lateralization of frontal and temporal coherences was observed. Nonetheless, there was no simple association of temporal lobe epileptic pathology that could be concluded from these two cases which appeared to be straightforward single sided temporal lobe epilepsy from the dEEG evaluation, and which have been seizure-free following temporal lobectomy.

Patient 2’s parents decided not to pursue surgical intervention. Yet the dEEG localization of both spikes and seizure onset pointed to a right temporal localization. With no indications of other epileptic foci, the dEEG localization has generally proven accurate for predicting both icEEG results and surgical outcome (Holmes et al., 2008, 2010b; Holmes, 2011). Nonetheless, Patient 2 showed a consistent cluster of left-frontal and temporal peak coherences, for the delta and theta bands, a pattern that seems discrepant with the more standard dEEG localization of spike and seizure source amplitudes.

Patient 3 was a difficult case, in that the primary findings pointed to the left superior medial frontal area, and there was a possible cortical anomaly in this area, and yet surgical resection of this area was only effective in improving, rather than eliminating, seizures. The largest discharges for both spikes and seizure onset were clearly localized to the left superior medial frontal area for Patient 3. There were subtle clues in the dEEG amplitude analysis that these discharges (for both spikes and the large discharge at seizure onset) were preceded by a source amplitude increase in the left temporal area. However, temporal lobe source activity is often associated with the early milliseconds of spike discharges in our experience, but it is still unclear whether this has clinical significance. It is worth noting that Patient 3 showed a tight left temporal coherence cluster, in contrast to more distributed coherences over the right hemisphere (Figure 8), in a mirror image of the pattern observed for Patient 1. Whether or not this restriction of hemispheric peak coherences to the temporal lobe could be taken as a signal of temporal lobe involvement in seizure onset is unclear without further studies.

Patient 4 is another example where early source amplitude (rather than coherence) changes are observed in the temporal lobe at the onset of spikes, but with uncertain clinical significance. In examining each of Patient 4’s several spike types (left inferior, right inferior), a left medial temporal source seemed to be the first indicator of the discharge. However, seizures appeared to show a midline onset, not only in the conventional EEG but in the dEEG as well. Furthermore, bilateral icEEG recordings showed apparent seizure onsets from both left and right midlines. The peak coherence patterns during spikes for Patient 4 showed left temporal and posterior clusters for delta and theta bands.

Although such clues are intriguing, the challenge for a novel method like cortical source coherence analysis will be to obtain validation from a number of converging perspectives, including normal and functional studies as well as epilepsy studies. We think it is impressive that the delta band of coherence shows apparently meaningful results in these analyses, and to a lesser extent theta band. Whereas the transient features of the spike amplitude involve spectral components in the delta and theta frequency bands, there is typically little power in the gamma band in the head surface EEG. Furthermore, gamma frequency of the EEG is often contaminated by muscle artifact. Nonetheless, although it is clearly important to consider artifactual explanations for the apparent EEG gamma patterns in high frequency analyses, it appears at the present time that meaningful coherence results can be obtained in this high frequency range in human subjects. The observation of high frequency oscillations in intracranial EEG at seizure onset (Kobayashi et al., 2010; Jacobs et al., 2011) makes it clear that high frequency features are an important target for non-invasive EEG analysis, and future research should examine the high gamma band (above 70 Hz).

A clear limitation of the present study is that we only examined EEG coherence around spike events rather than around seizure onset. Although interictal events are often localizing for seizure onset, there are very likely dynamics of cortical synchronization that can only be studied in relation to seizure onset.

Another limitation of the present study is that we grouped the oriented cortical sources into Brodmann areas, primarily for computational convenience. A goal for research on oriented cortical sources must be to implement the high performance computing necessary to examine the full cortical source coherence matrix, applying not only statistical analysis and decomposition but also effective visualization methods. Recent advances in diffusion imaging tractography (Scherrer and Warfield, 2010) have made it possible to compute a full set of likely cerebral fiber tracts (*N* = 15 million), and to align them with both subcortical structures and the cortical patch tessellation implemented in the present study. Anatomically specific measures of cortical electrophysiology will continue to bring important tools to the study of epilepsy in the near future. The challenge will be to apply these tools to the study of epileptic networks in order to understand their clinical significance.

## Conclusion

EEG coherence provides a measure of the covariance among signals that resolves the frequency features of that covariance. In the present research, coherence was computed among cortical source waveforms, localized to the oriented cortical surface with detailed head models for individual patients. The improved source analysis with anatomical constraints promises new insights into the network properties that may be altered in epilepsy. The first step in these analyses was localizing the amplitude onset of the spike with 256 dEEG, which proved useful in predicting the onset of the seizure in each case. The utility of dEEG spike localization was verified by dEEG and icEEG analyses of seizure onset, and was confirmed in several cases by success of surgical resection. Whether cortical source coherence analysis adds to the clinical utility of spike amplitude localization remains to be seen. Perhaps the most intriguing observation was the frequent pattern of strong coherence centered on temporal lobe structures in several patients. Understanding the clinical significance of this pattern will require further studies of seizure onset, as well as contrast analyses with normal individuals and with apparently normal EEG intervals in the epileptic patients.

## Conflict of Interest Statement

The authors declare that the research was conducted in the absence of any commercial or financial relationships that could be construed as a potential conflict of interest.
